# Supplementary material for: Efficient Genome Editing Using ‘NanoMEDIC’ AsCas12a-VLPs Produced with Pol II-Transcribed crRNA
Source: Int J Mol Sci. 2024 Nov 27;25(23):12768. doi: 10.3390/ijms252312768 (PMC11641575; doi:10.3390/ijms252312768)
Supplement: Supplementary file 1 [file ijms-25-12768-s001.zip › ijms-3321763-supplementary.pdf]

Supplementary Information

Efficient genome editing with AsCas12a-VLPs produced with Pol II promoter-transcribed crRNA

Sofiia E. Borovikova, Mikhail V. Shepelev, Dmitriy V. Mazurov and Natalia A. Kruglova

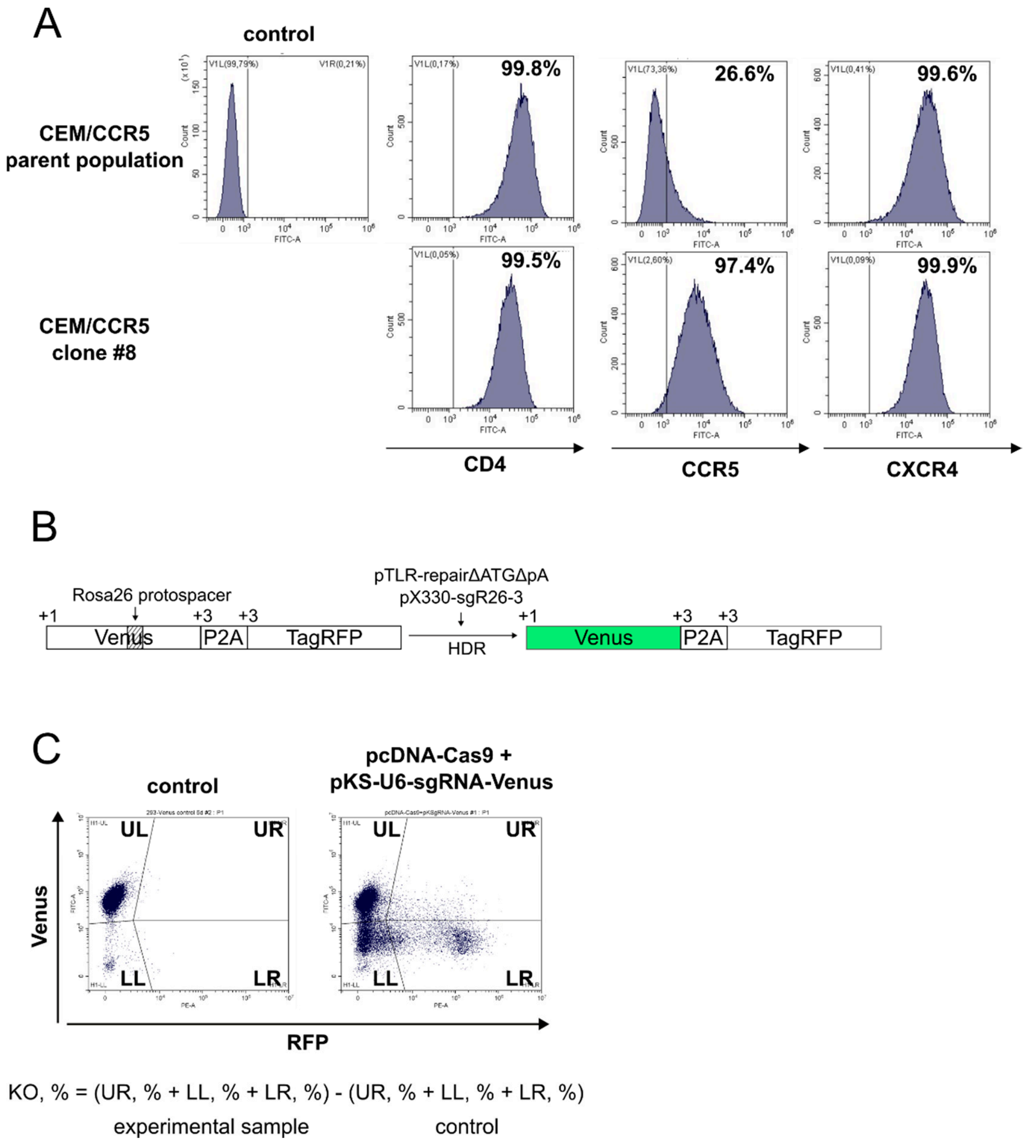

Supplementary Figure S1. (**A**) Surface level of the CD4, CCR5, and CXCR4 receptors on the population or clone #8 of CEM/CCR5 cells. Cells were stained with the corresponding mouse antibodies against CD4, CCR5, or CXCR4, followed by staining with secondary antibodies against mouse IgG labelled with Alexa 488. (**B**) Scheme of generation of 293-Venus reporter cells. TLR5 reporter construct was knocked-in into the *AAVS1* locus of HEK293 cells and then edited using pTLR-repairΔATGΔpA donor to produce cells that express Venus, but not TagRFP. (**C**) Example of the *Venus* reporter editing in 293-Venus clone #8 cells. Representative dot plots show non-transfected control 293-Venus clone #8 cells and the cells transfected with plasmids pcDNA3.3-hSpCas9 and pKS-U6-sgRNA targeting *Venus*. The formula for calculation of the knockout level is presented.

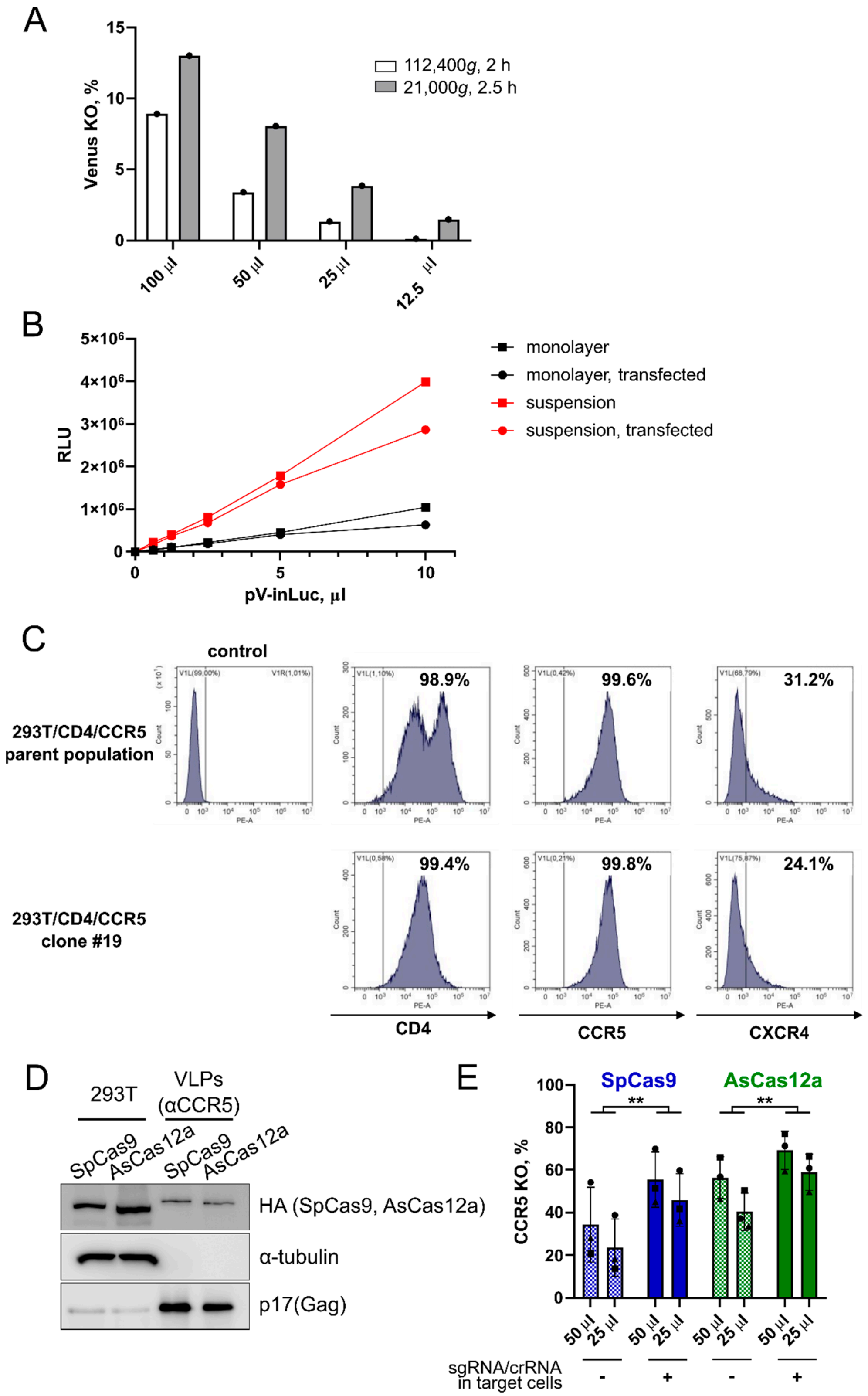

Supplementary Figure S2. (**A**) Flow cytometry analysis of the *Venus* knockout level in 293-Venus clone #8 cells induced by VLPs with SpCas9 that were concentrated either by ultracentrifugation (112,400*g*, 2 h) or by centrifugation at 21,000*g*, 2.5 h. (**B**) Infectivity levels of pseudoviral particles carrying inLuc luciferase as a reporter (pV-inLuc, [57]) added to target 293T/CD4/CCR5 clone #19 cells in suspension (red curves) or in monolayer (black curves) and measured 48h after transduction. Squares correspond to target cells

preliminary transfected with GFP plasmid; circles correspond to control cells without transfection. RLU, relative luciferase units. (**C**) Surface level of the CD4, CCR5, and CXCR4 receptors on the population or clone #19 of 293T/CD4/CCR5 cells. Cells were stained with the corresponding mouse antibodies against CD4, CCR5, or CXCR4, followed by staining with secondary antibodies against mouse IgG labelled with Alexa 546. (**D**) Representative Western blot evaluating the nuclease content in lysates of 293T producer cells and VLPs. (**E**) Flow cytometry analysis of the *CCR5* knockout level in 293T/CD4/CCR5 clone #19 mediated by VLPs with AsCas12a or SpCas9. Shaded bars correspond to target cells preliminary transfected with the plasmid encoding gRNA, dashed bars depict target cells transfected with the plasmid coding for control gRNA. Results from three independent experiments are shown as individual data points and as mean ± standard deviation; different symbols correspond to independent experiments. Mean values were compared by three-way ANOVA (with VLP dose, presence of crRNA in target cells, and nuclease type as factors) with subsequent Sidak's multiple comparison test (**) $p < 0.01$.

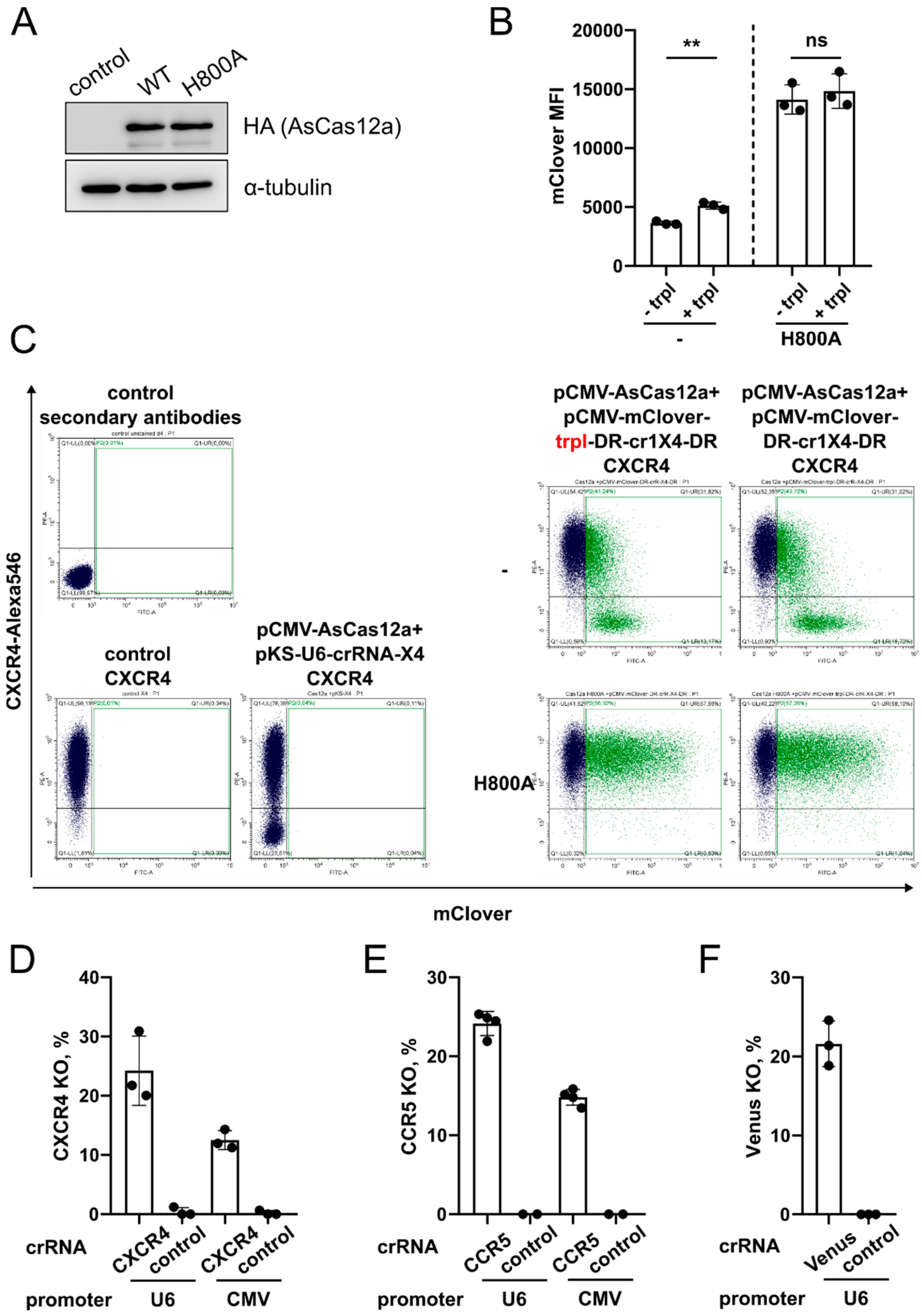

Supplementary Figure S3. (**A**) Representative Western blot evaluating the nuclease content in lysates of 293T producer cells. (**B**), (**C**) Flow cytometry analysis of the mClover MFI in CEM/CCR5 cells electroporated with plasmids encoding AsCas12a or AsCas12a H800A and pCMV-based crRNA plasmid with or without Triplex. Mean fluorescence intensity (MFI) values from three independent experiments (**B**) and representative dot plots (**C**) with the mClover+ gate (green) are shown. (**D**), (**E**), (**F**) Flow cytometry analysis of the *CXCR4* (**D**), *CCR5* (**E**), and *Venus* (**F**) knockout levels in corresponding cells mediated by AsCas12a and crRNA expressed under the control of U6 or CMV promoter, control samples included irrelevant crRNA. Mean values were compared by unpaired t-test with Bonferroni correction (*) $p < 0.01$ (**C**).

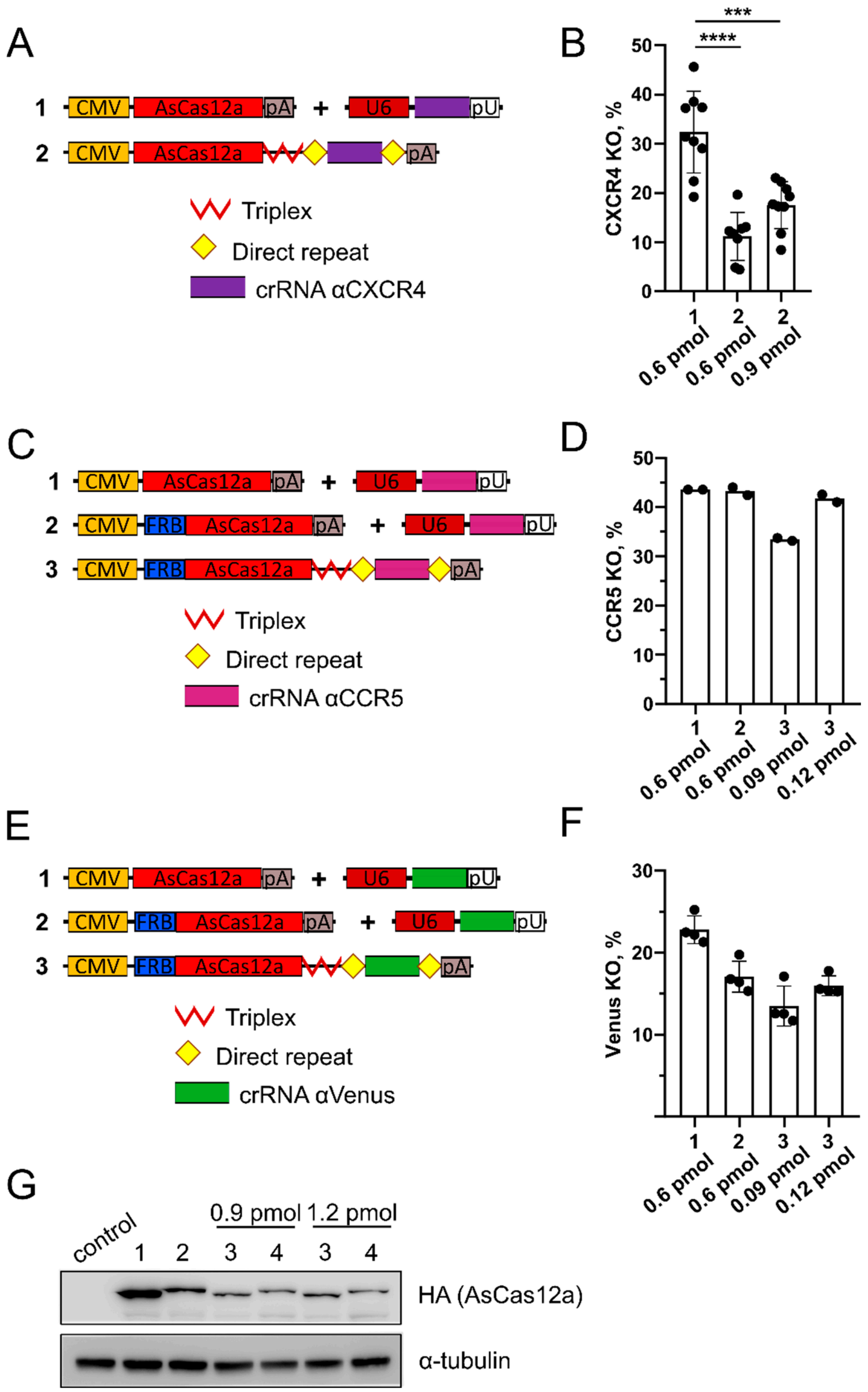

Supplementary Figure S4. (**A**), (**C**), (**E**) Schemes of plasmids encoding AsCas12a and crRNA. U6 – U6 promoter, pA – SV40 virus polyadenylation signal, pU – poly(U) transcription termination signal. (**B**) Flow cytometry analysis of the *CXCR4* knockout level in CEM/CCR5 cells electroporated with one of the plasmid variants shown in (**A**). (**D**) Flow cytometry analysis of the *CCR5* knockout level in 293T/CD4/CCR5 clone #19 cells transfected with one of the plasmid variants shown in (**C**). (**F**) Flow cytometry analysis of the *Venus* knockout level in 293-Venus clone #9 cells transfected with one of the plasmid variants shown in (**E**). (**G**) Representative Western blot evaluating the level of AsCas12a and FRB-AsCas12a variants in transfected 293T cells. Numbers refer to constructs shown in Figure 4C. Mean values were compared by one-way ANOVA for independent samples with subsequent Tukey's test for multiple comparisons (***) $p < 0.001$, (****) $p < 0.0001$.

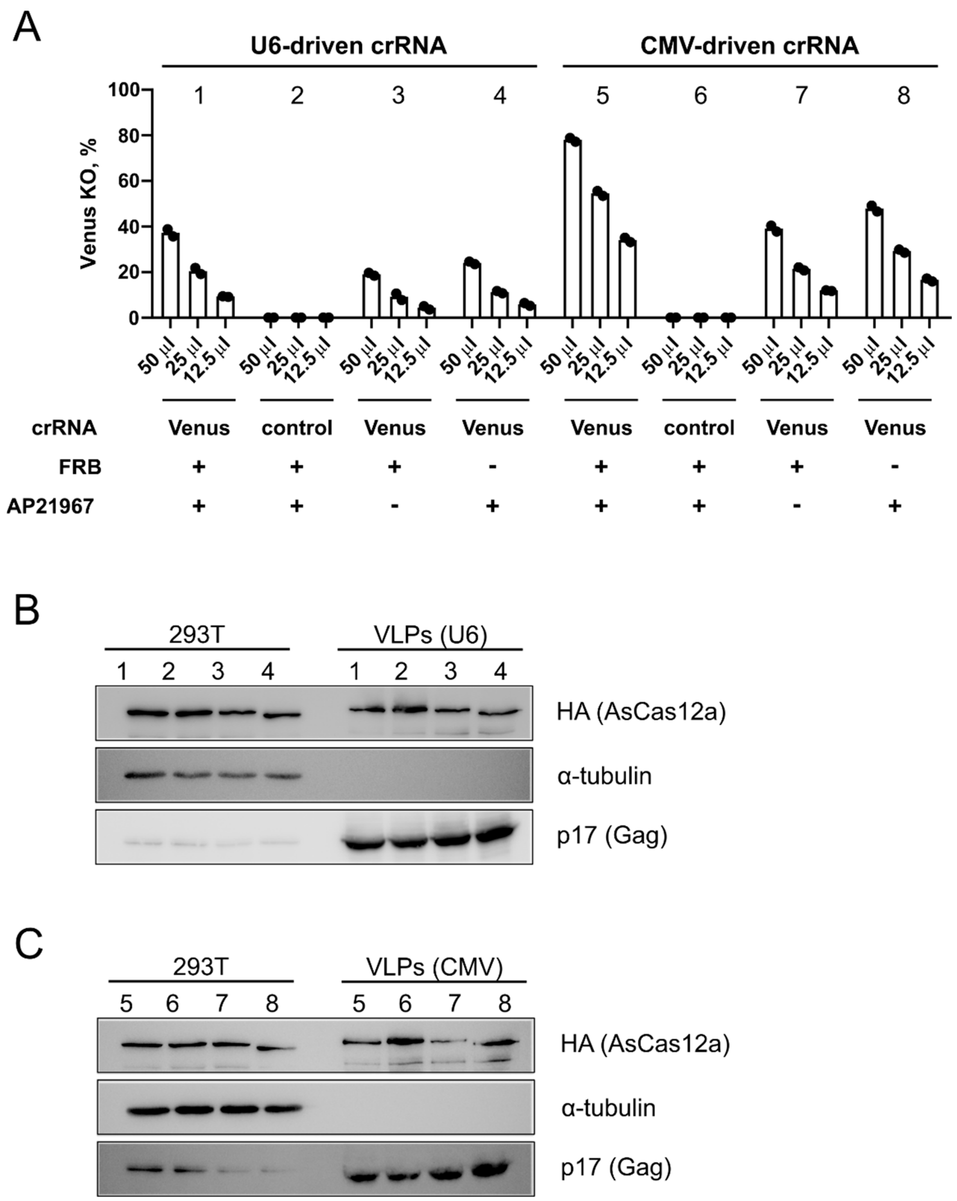

Supplementary Figure S5. (**A**) Flow cytometry analysis of the *Venus* knockout levels in 293-Venus clone #8 cells that were transduced with VLPs produced with U6- or CMV-promoter driven crRNA. Results of two independent transduction experiments with the same VLP preparations are shown. (**B**), (**C**) Western blot analysis of the AsCas12a nuclease content in lysates of 293T producer cells and VLPs targeting *Venus* and produced with U6- (**B**) or CMV- (**C**) driven crRNA.

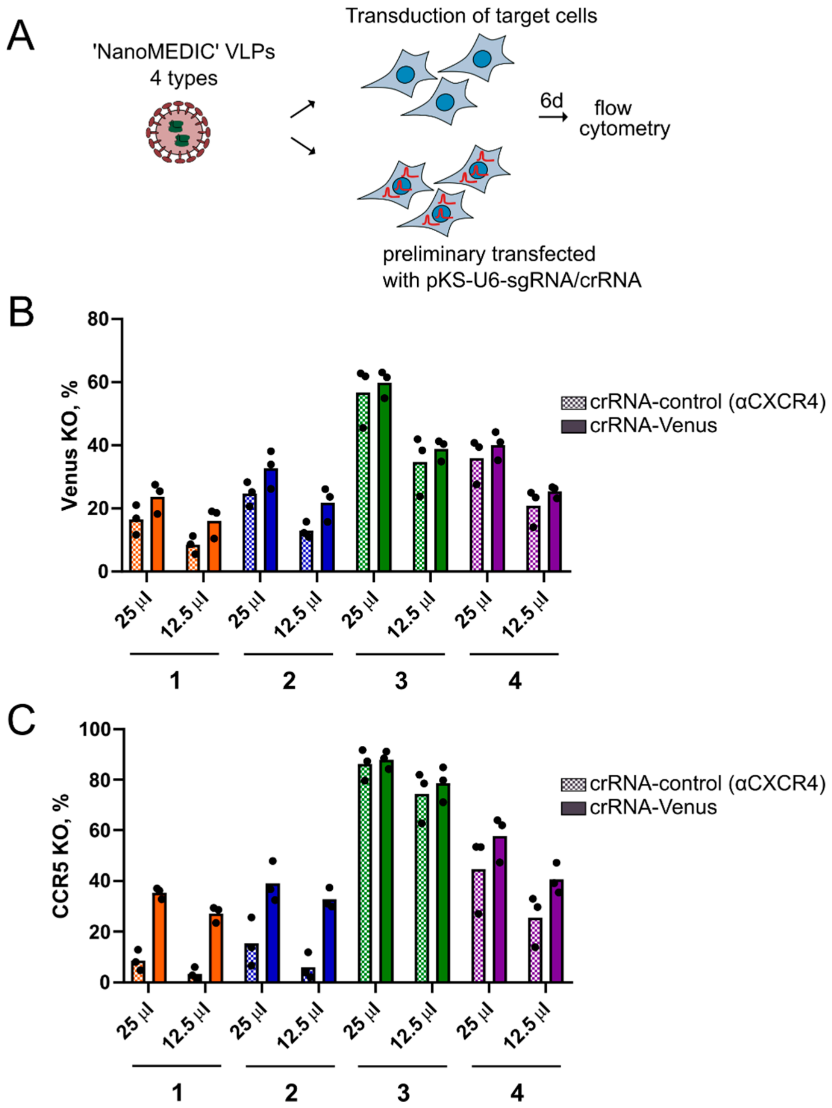

Supplementary Figure S6. (**A**) Scheme of VLP transduction experiment with target cells expressing crRNA. Flow cytometry analysis of the *Venus* (**B**) and *CCR5* (**C**) knockout levels in 293-Venus clone #8 and 293T/CD4/CCR5 clone #19 cells, respectively. Cells were transduced with VLP preparations #1-4 that are shown in the Figure 6A.

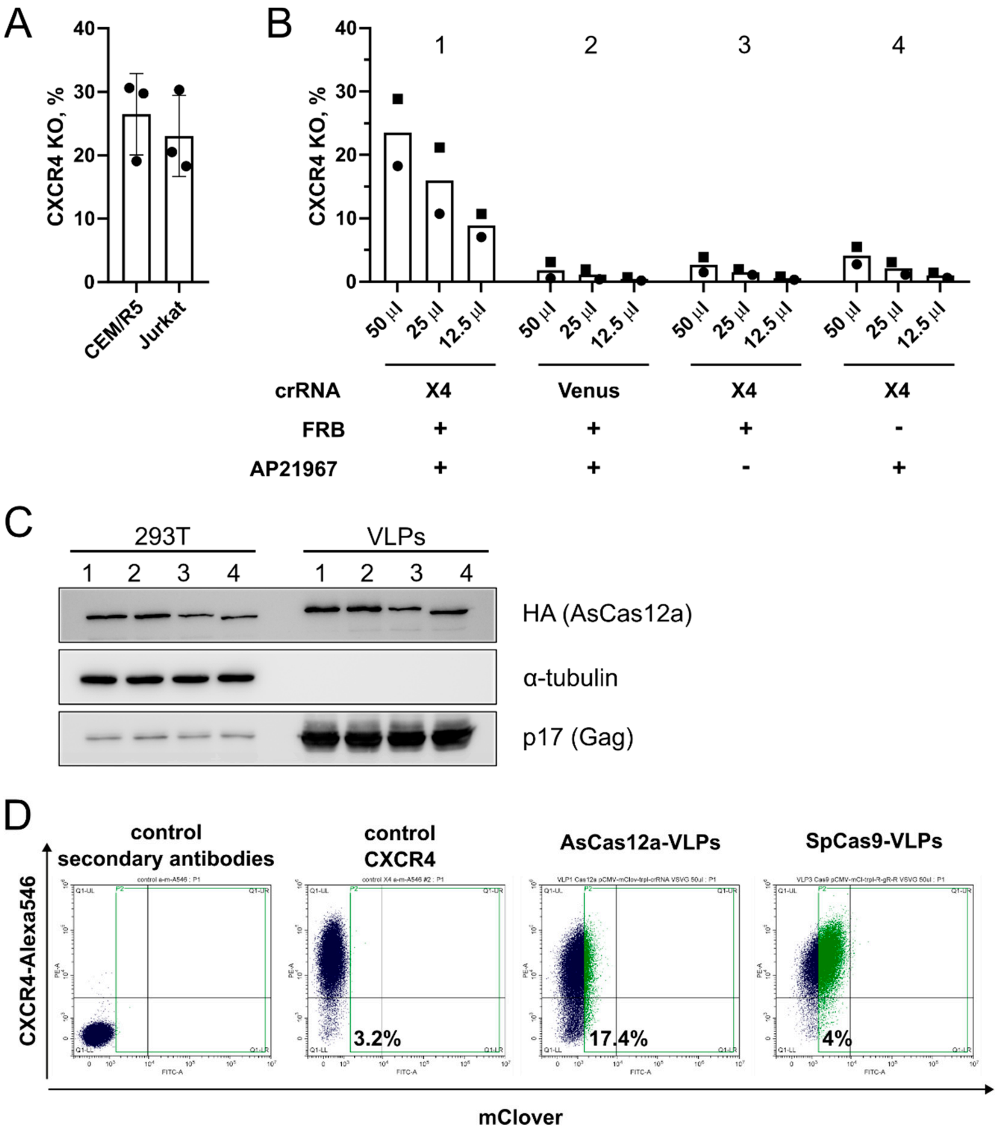

Supplementary Figure S7. (**A**) Flow cytometry analysis of the *CXCR4* knockout levels in CEM/CCR5 and Jurkat T cells electroporated with pcDNA3.3-hSpCas9 and pKS-U6-sgRNA. (**B**) Flow cytometry analysis of the *CXCR4* knockout levels in Jurkat T cells that were transduced with VLPs produced with pCMV-FRB-AsCas12a and pCMV-mClover-trpl-DR-cr1X4-DR (design #3 according to the Figure 6A) and control VLP samples (with crRNA targeting *Venus*, produced without AP21967 or with AsCas12a without FRB). Data points for independent experiments #1 and #2 are shown by circles and squares, respectively. (**C**) Western blot results evaluating the nuclease content in lysates of 293T producer cells and VLPs #1-4 (correspond to the results that are shown in (**B**)). (**D**) Flow cytometry data of the *CXCR4* knockout levels in Jurkat T cells that were transduced with AsCas12a-VLPs #3 or SpCas9-VLPs produced with pCMV-mClover-trpl-Rib-sg1X4-Rib. Figures represent CXCR4− cells, green gate shows mClover+ cells.

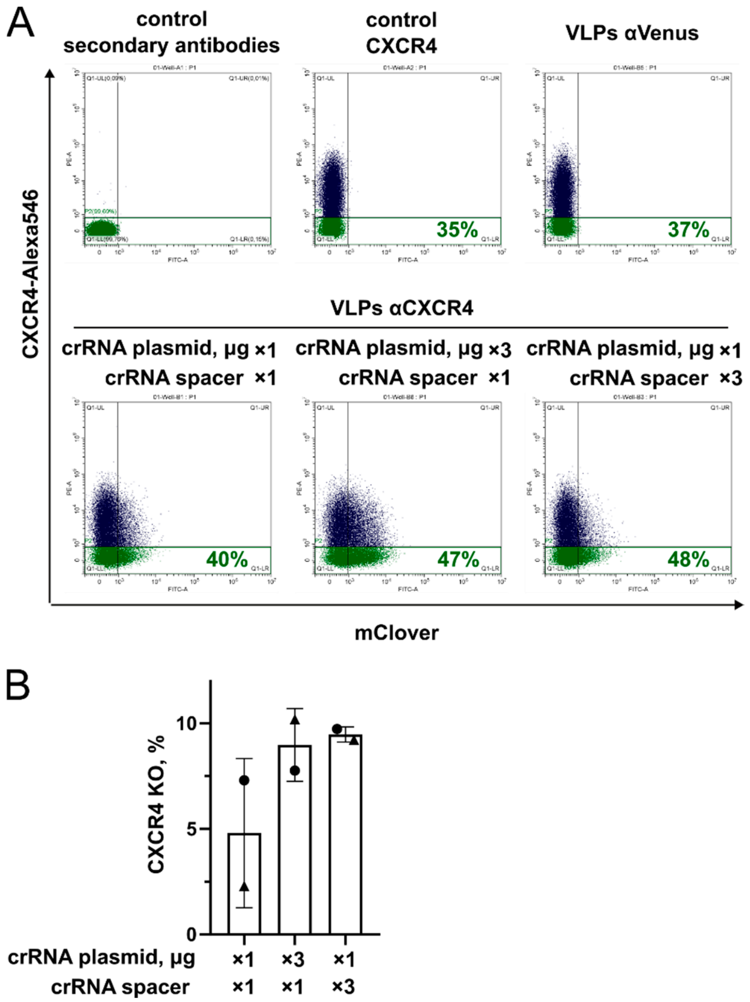

Supplementary Figure S8. (**A**) Flow cytometry analysis of the *CXCR4* knockout levels in primary CD4+ T cells that were transduced with VLPs #3 (referred to the design #3, Figure 6A). The plasmid pCMV-mClover-trpl-DR-cr1X4-DR coding for crRNA was used at the dose 1.66 μg (crRNA plasmid, μg ×1, crRNA spacer ×1) or the tripled dose (crRNA plasmid, μg ×3, crRNA spacer ×1); alternatively, the plasmid the plasmid pCMV-mClover-trpl-DR-cr3X4-DR coding for 3 spacers (crRNA spacer ×3) was used. pCMV-eBFP2 -trpl-DR-cr1Venus-DR was used as a control. Dot plots for the donor #1 (**A**) and summary results for two donors (**B**) are shown.

Supplementary Table S1. Sequences of protospacers for target loci.

Target locus	Nuclease	protospacer, PAM	Source
Venus	SpCas9	cgtcgccgtccagctcgaccAGG	sgRNA #g1 [32]
Venus	AsCas12a	TTTAcgtcgccgtccagctcgacc	crRNA #g1 [32]
CXCR4	SpCas9	cacttcagataactacaccgAGG	sgRNA CXCR4 exon-2 [24]
CXCR4-1	AsCas12a	TTTGcagatatacacttcagataacta	crRNA-1 [26]
CXCR4-2	AsCas12a	TTTGggaacttcctatgcaaggcagtc	crRNA-2 [27]
CXCR4-3	AsCas12a	TTTCttcacggaaacagggttccttca	crCXCR4_10 [28]
CCR5-1	SpCas9	cagcatagtgagcccagaagGGG	[30]
CCR5-2	SpCas9	tgacatcaattattatacatCGG	[31]
CCR5-1	AsCas12a	TTTAggattcccgagtagcagatgacc	crRNA-1 [29]
CCR5-2	AsCas12a	TTTAtcaggatgaggatgaccagcatg	crRNA-3 [29], crRNA-4 [27]
CCR5-3	AsCas12a	TTTGgaaatacaatgtgtcaactcttg	crRNA-5 [27]
AAVS1-2	SpCas9	gtcaccaatcctgtccctagTGG	[25]
Rosa26-3	SpCas9	cagtctttctagaagatgggCGG	This study, designed using ChopChop (https://chopchop.cbu.uib.no/)

Supplementary Table S2. Oligonucleotides used for cloning.

Oligonucleotide	Sequence	Description
5′-sgRNA-Venus	caccgcgtcgccgtccagctcgacc	Cloning the plasmid pKS-U6-sgRNA targeting *Venus*
3′-sgRNA-Venus	aaacggtcgagctggacggcgacgc	Cloning the plasmid pKS-U6-sgRNA targeting *Venus*
5′-crRNA-Venus	tagatcgtcgccgtccagctcgacc	Cloning the plasmid pKS-U6-crRNA targeting *Venus*
3′-crRNA-Venus	aaaaggtcgagctggacggcgacga	Cloning the plasmid pKS-U6-crRNA targeting *Venus*
5′-sgRNA-CXCR4	caccgcacttcagataactacaccg	Cloning the plasmid pKS-U6-sgRNA targeting *CXCR4*
3′-sgRNA-CXCR4	aaaccggtgtagttatctgaagtgc	Cloning the plasmid pKS-U6-sgRNA targeting *CXCR4*
5′-crRNA-CXCR4-1	tagatcagatatacacttcagataacta	Cloning the plasmid pKS-U6-crRNA targeting *CXCR4*
3′-crRNA-CXCR4-1	aaaatagttatctgaagtgtatatctga	Cloning the plasmid pKS-U6-crRNA targeting *CXCR4*
5′-crRNA-CXCR4-2	tagatggaacttcctatgcaaggcagtc	Cloning the plasmid pKS-U6-crRNA targeting *CXCR4*
3′-crRNA-CXCR4-2	aaaagactgccttgcataggaagttcca	Cloning the plasmid pKS-U6-crRNA targeting *CXCR4*
5′-crRNA-CXCR4-3	tagatttcacggaaacagggttccttca	Cloning the plasmid pKS-U6-crRNA targeting *CXCR4*
3′-crRNA-CXCR4-3	aaaatgaaggaaccctgtttccgtgaaa	Cloning the plasmid pKS-U6-crRNA targeting *CXCR4*
5′-sgRNA-CCR5-1	caccgtgacatcaattattatacat	Cloning the plasmid pKS-U6-sgRNA targeting *CCR5*
3′-sgRNA-CCR5-1	aaacatgtataataattgatgtcac	Cloning the plasmid pKS-U6-sgRNA targeting *CCR5*
5′-sgRNA-CCR5-2	caccgcagcatagtgagcccagaag	Cloning the plasmid pKS-U6-crRNA targeting *CCR5*
3′-sgRNA-CCR5-2	aaaccttctgggctcactatgctgc	Cloning the plasmid pKS-U6-crRNA targeting *CCR5*
5′-crRNA-CCR5-1	tagatggattcccgagtagcagatgacc	Cloning the plasmid pKS-U6-crRNA targeting *CCR5*
3′-crRNA-CCR5-1	aaaaggtcatctgctactcgggaatcca	Cloning the plasmid pKS-U6-crRNA targeting *CCR5*
5′-crRNA-CCR5-2	tagattcaggatgaggatgaccagcatg	Cloning the plasmid pKS-U6-crRNA targeting *CCR5*
3′-crRNA-CCR5-2	aaaacatgctggtcatcctcatcctgaa	Cloning the plasmid pKS-U6-crRNA targeting *CCR5*
5′-crRNA-CCR5-3	tagatgaaatacaatgtgtcaactcttg	Cloning the plasmid pKS-U6-crRNA targeting *CCR5*
3′-crRNA-CCR5-3	aaaacaagagttgacacattgtatttca	Cloning the plasmid pKS-U6-crRNA targeting *CCR5*
sgRosa26-3_F	cacccagtctttctagaagatggg	Cloning the plasmid pX330-sgR26-3
sgRosa26-3_R	aaaccccatcttctagaaagactg	Cloning the plasmid pX330-sgR26-3
sgAAVS-2_F	caccgtcaccaatcctgtccctag	Cloning the plasmid pX458-sgAAVS2
sgAAVS-2_R	aaacctagggacaggattggtgac	Cloning the plasmid pX458-sgAAVS2

5′-VenRI	aaaaagcttgaattcgtgagcaagggcgagg	Construction of pTLR-donor-ΔATGΔpolyA
3′-VenSp	aaaactagtctacttgtacagctcgtc	Construction of pTLR-donor-ΔATGΔpolyA
5′-crRNA-scaffold	caccgtaatttctactcttgtagatggtcttcgagaagaccttttttt	Replacement of SpCas9 gRNA scaffold in pKS-U6-gRNA-BB vector with AsCas12a crRNA scaffold
3′-crRNA-scaffold	ctagaaaaaaaggtcttctcgaagaccatctacaagagtagaaattac	Replacement of SpCas9 gRNA scaffold in pKS-U6-gRNA-BB vector with AsCas12a crRNA scaffold
5′-NotI-3xHA-1	ggccgcccccaagaagaagaggaaggtgggatcctacccatacgatgttccagattacgcttatccctacgacgtg	Cloning of 3xHA tag to the C-terminus of AsCas12a in pCMV-AsCas12a-2xNLS
5′-NotI-3xHA-2	cctgattatgcatacccatatgatgtccccgactatgcc**tga**c	Cloning of 3xHA tag to the C-terminus of AsCas12a in pCMV-AsCas12a-2xNLS
3′-XmaI-3xHA-1	catcgtatgggtaggatcccaccttcctcttcttcttgggggc	Cloning of 3xHA tag to the C-terminus of AsCas12a in pCMV-AsCas12a-2xNLS
3′-XmaI-3xHA-2	ccgggtcaggcatagtcggggacatcatatgggtatgcataatcaggcacgtcgtagggataagcgtaatctggaa	Cloning of 3xHA tag to the C-terminus of AsCas12a in pCMV-AsCas12a-2xNLS
5′-XbaI-FRB	actctagagggccaccatggctgctagaatcctctggcatgagatgtg	Cloning of FRB to the N-terminus of AsCas12a in pCMV-AsCas12a
3′-FRB-NheI-Cas12	ccctcgaactgtgtgccgctagcctttgagattcgtcggaaca	Cloning of FRB to the N-terminus of AsCas12a in pCMV-AsCas12a
5′-Cas12	ggcacacagttcgaggg	Cloning of FRB to the N-terminus of AsCas12a in pCMV-AsCas12a
3′-Cas12a-BlpI	gaaggcctcgctcagct	Cloning of FRB to the N-terminus of AsCas12a in pCMV-AsCas12a
5′-NotI-NLS-NheI-FRB	cggcggccgcccccaagaagaagaggaaggtggctagcatcctctggcatgagatgtg	Cloning of FRB to the C-terminus of AsCas12a in pCMV-AsCas12a
3′-FRB-BamHI	gcggatccctttgagattcgtcggaacac	Cloning of FRB to the C-terminus of AsCas12a in pCMV-AsCas12a
5′-H800A	caggatgaagaggat**GGC**agcccggctggga	Generation of AsCas12a H800A RNase-deficient mutant
3′-H800A	tcccagccgggctgccatcctcttcatcctg	Generation of AsCas12a H800A RNase-deficient mutant
5′-MCS-NM	gtacaagtaagaattcatcgatgcccgggtatacgcgttgtaccggtaac	Cloning of the plasmid pCMV-mClover-trpl-Rib-sg1X4-Rib
3′-MCS-NM	tcgagttaccggtacaacgcgtatacccgggcatcgatgaattcttactt	Cloning of the plasmid pCMV-mClover-trpl-Rib-sg1X4-Rib
5′-XmaI-Trpl	ccgggattcgtcagtagggttgtaaaggttttctttcctgagaaaacaaccttttgttttctcaggttttgctttttggcctttccctagctttaaaaaaaaaaaagcaaaa	Cloning of the plasmid pCMV-mClover-trpl-Rib-sg1X4-Rib
3′-MluI-Trpl	cgcgttttgcttttttttttaaagctagggaaaggccaaaaagcaaaacctgagaaaacaaaaggttgttttctcaggaaaagaaaaaccctttacaaccctactgacgaatc	Cloning of the plasmid pCMV-mClover-trpl-Rib-sg1X4-Rib
5′-HH-X4	cgacgcgtctgatgagtccgtgaggacgaaacgagtaagctcgtccacttcagataactacaccg	Cloning of the plasmid pCMV-mClover-trpl-Rib-sg1X4-Rib
3′-X4-HDV	gactcgagtcccattcgccatgccgaagcatgttgcccagccggcgccagcgaggag	Cloning of the plasmid pCMV-mClover-trpl-Rib-sg1X4-Rib

	gctgggaccatgccggccaaaaaaagcaccgactcggt	
5′-crRNA-X4-crRNA	cgcgtaatttctactcttgtagatttcacggaaacagggttccttcaaatttctactcttgtagatac	Cloning of the construct with CMV-driven crRNA expression
3′-crRNA-X4-crRNA	tcgagtatctacaagagtagaaatttgaaggaaccctgtttccgtgaaatctacaagagtagaaatta	Cloning of the construct with CMV-driven crRNA expression
5′-crRNA-R5-crRNA	cgcgtaatttctactcttgtagattcaggatgaggatgaccagcatgaatttctactcttgtagatac	Cloning of the construct with CMV-driven crRNA expression
3′-crRNA-R5-crRNA	tcgagtatctacaagagtagaaattcatgctggtcatcctcatcctgaatctacaagagtagaaatta	Cloning of the construct with CMV-driven crRNA expression
5′-crRNA-Venus-crRNA	cgcgtaatttctactcttgtagatcgtcgccgtccagctcgaccaatttctactcttgtagatac	Cloning of the construct with CMV-driven crRNA expression
3′-crRNA-Venus-crRNA	tcgagtatctacaagagtagaaattggtcgagctggacggcgacgatctacaagagtagaaatta	Cloning of the construct with CMV-driven crRNA expression
5′-PspXI-XbaI-eBFP2	cgcaggctcgagttctagaaccaccatggtgagcaagggcga	Replacement of mClover sequence in pCMV-mClover-DR-crVenus-DR with eBFP2 sequence
3′-eBFP2-ClaI	cgggcatcgattcacttgtacagctcgtcca	Replacement of mClover sequence in pCMV-mClover-DR-crVenus-DR with eBFP2 sequence
5′-insert-MluI	cgcgtctcaccgaggcagttccataggatggcaagatcctggtattggctagcga	Cloning of 50 nt insert between Triplex and the first direct repeat in pCMV-AsCas12a-trpl-DR-crX4-DR
3′-insert	cgcgtcgctagccaataccaggatcttgccatcctatggaactgcctcggtgaga	Cloning of 50 nt insert between Triplex and the first direct repeat in pCMV-AsCas12a-trpl-DR-crX4-DR
5′-Mlu-DR-X4-DR-BbsI-Acc65I	cgcgtaatttctactcttgtagatttcacggaaacagggttccttcaaatttctactcttgtagataggtcttcactcgagtg	Cloning of crRNA array with several identical spacers in pCMV-AsCas12a-trpl-DR-crX4-DR
3′-Mlu-DR-X4-DR-BbsI-Acc65I	gtaccactcgagtgaagacctatctacaagagtagaaatttgaaggaaccctgtttccgtgaaatctacaagagtagaaatta	Cloning of crRNA array with several identical spacers in pCMV-AsCas12a-trpl-DR-crX4-DR
5′-DR-X4-DR-BbsI-Acc65I	agatttcacggaaacagggttccttcaaatttctactcttgtagataggtcttcactcgagtg	Cloning of crRNA array with several identical spacers in pCMV-AsCas12a-trpl-DR-crX4-DR
3′-DR-X4-DR-BbsI-Acc65I	gtaccactcgagtgaagacctatctacaagagtagaaatttgaaggaaccctgtttccgtgaa	Cloning of crRNA array with several identical spacers in pCMV-AsCas12a-trpl-DR-crX4-DR
5′-DR-X4-DR-Acc65I	agatttcacggaaacagggttccttcaaatttctactcttgtagatactcgagtg	Cloning of crRNA array with several identical spacers in pCMV-AsCas12a-trpl-DR-crX4-DR
3′-DR-X4-DR-Acc65I	gtaccactcgagtatctacaagagtagaaatttgaaggaaccctgtttccgtgaa	Cloning of crRNA array with several identical spacers in pCMV-AsCas12a-trpl-DR-crX4-DR
5′-Mlu-4tDR-X4-4tDR-Acc65I	cgcgt**tttt**aatttctactcttgtagatttcacggaaacagggttccttca**tttt**aatttctactcttgtagatactcgagtg	Cloning of crRNA array with several identical spacers and optimized direct repeats in pCMV-AsCas12a-trpl-DR-crX4-DR

3′-Mlu-4tDR-X4-4tDR-Acc65I	gtaccactcgagtatctacaagagtagaaattaaaatgaaggaaccctgtttccgtgaaatctacaagagtagaaattaaaaa	Cloning of crRNA array with several identical spacers and optimized direct repeats in pCMV-AsCas12a-trpl-DR-crX4-DR
5′-Mlu-4tDR-X4-4tDR-BbsI-Acc65I	cgcg**tttt**aatttctactcttgtagatttcacggaaacagggttccttca**tttt**aatttctactcttgtagataggtcttcactcgagtg	Cloning of crRNA array with several identical spacers and optimized direct repeats in pCMV-AsCas12a-trpl-DR-crX4-DR
3′-Mlu-4tDR-X4-4tDR-BbsI-Acc65I	gtaccactcgagtgaagacctatctacaagagtagaaattaaaatgaaggaaccctgtttccgtgaaatctacaagagtagaaattaaaaa	Cloning of crRNA array with several identical spacers and optimized direct repeats in pCMV-AsCas12a-trpl-DR-crX4-DR
5′-4tDR-X4-4tDR-BbsI-Acc65I	agatttcacggaaacagggttccttca**tttt**aatttctactcttgtagataggtcttcactcgagtg	Cloning of crRNA array with several identical spacers and optimized direct repeats in pCMV-AsCas12a-trpl-DR-crX4-DR
3′-4tDR-X4-4tDR-BbsI-Acc65I	gtaccactcgagtgaagacctatctacaagagtagaaattaaaatgaaggaaccctgtttccgtgaa	Cloning of crRNA array with several identical spacers and optimized direct repeats in pCMV-AsCas12a-trpl-DR-crX4-DR
5′-4tDR-X4-4tDR-Acc65I	agatttcacggaaacagggttccttca**tttt**aatttctactcttgtagatactcgagtg	Cloning of crRNA array with several identical spacers and optimized direct repeats in pCMV-AsCas12a-trpl-DR-crX4-DR
3′-4tDR-X4-4tDR-Acc65I	gtaccactcgagtatctacaagagtagaaattaaaatgaaggaaccctgtttccgtgaa	Cloning of crRNA array with several identical spacers and optimized direct repeats in pCMV-AsCas12a-trpl-DR-crX4-DR
5′-3R5	cttaagaatttctactct	Cloning of an array of three AsCas12a crRNAs against *CCR5* flanked by direct repeats.
3′-3R5	ctcgagtatctacaag	Cloning of an array of three AsCas12a crRNAs against *CCR5* flanked by direct repeats.

Supplementary Table S3. Plasmid constructs obtained and/or used in this study.

Plasmid	Source	Description
pAAVS-TLR	#64215, Addgene [25]	Generation of HEK293-TLR5 reporter cell line, donor plasmid
pX458-sgAAVS2	This study	Generation of HEK293-TLR5 reporter cell line, plasmid encoding Cas9 and sgRNA against *AAVS1*
pX330-sgR26-3	This study	Generation of 293-Venus reporter cell line, plasmid encoding Cas9 and sgRNA against mouse *Rosa26*
pTLR-donor-ΔATGΔpolyA	This study	Generation of 293-Venus reporter cell line, donor plasmid
pKS-U6-gRNA-BB	[24]	BbsI-mediated cloning of sgRNAs for SpCas9
pKS-U6-crRNA-BB	This study	BbsI-mediated cloning of crRNAs for AsCas12a
pX330	#42230, Addgene [52]	BbsI-mediated cloning of sgRNAs for SpCas9
pX458	#48138, Addgene [51]	BbsI-mediated cloning of sgRNAs for SpCas9
pcDNA3.3-hSpCas9	#41815, Addgene [54]	Plasmid expressing SpCas9
pCMV-mClover-trpl-Rib-sg1X4-Rib	This study	CMV-promoter based plasmid encoding mClover which is followed by Triplex and SpCas9 sgRNA against *CXCR4* flanked by HH and HDV ribozymes according to Gee at al. [11]
pCMV-mClover-trpl-DR-cr1X4-DR	This study	CMV-promoter based plasmid encoding mClover which is followed by Triplex and AsCas12a crRNA against *CXCR4* flanked by direct repeats
pCMV-mClover-trpl-DR-cr1R5-DR	This study	CMV-promoter based plasmid encoding mClover which is followed by Triplex and AsCas12a crRNA against *CCR5* flanked by direct repeats
pCMV-eBFP2-trpl-DR-cr1Venus-DR	This study	CMV-promoter based plasmid encoding eBFP2 which is followed by Triplex and AsCas12a crRNA against *Venus* flanked by direct repeats
pU6-(BbsI)-CBh-Cas9-T2A-BFP	#64323, Addgene [25]	Plasmid for cloning eBFP2 sequence to produce pCMV-eBFP2-trpl-DR-crVenus-DR
pET-28b-T7-henAsCas12a-HF1(E174R/N282A/S542R/K548R)-NLS(nucleoplasmin)-6xHis	#114073, Addgene [33]	The plasmid from which the sequence of AsCas12a was cloned
pCMV-AsCas12a-2xNLS	This study	Plasmid encoding AsCas12a with 2xNLS
pCMV-AsCas12a-2xNLS-3xHA (=pCMV-AsCas12a)	This study	Plasmid encoding AsCas12a with 2xNLS and 3xHA and later referred to as pCMV-AsCas12a
pCMV-FRB-AsCas12a	This study	Plasmid encoding AsCas12a with 2xNLS and 3xHA and fused to the FRB domain at the N-terminus
pCMV-AsCas12a-FRB	This study	Plasmid encoding AsCas12a with 2xNLS and 3xHA and fused to the FRB domain at the C-terminus

pCMV-AsCas12a-H800A	This study	Plasmid encoding AsCas12a-H800A with 2xNLS and 3xHA (RNase-deficient mutant)
pCMV-AsCas12a-trpl-DR-cr1X4-DR	This study	Single plasmid encoding AsCas12a with 2xNLS which is followed by Triplex and AsCas12a crRNA against *CXCR4* flanked by direct repeats
pCMV-AsCas12a-trpl-ins_50nt-DR-cr1X4-DR	This study	Single plasmid encoding AsCas12a with 2xNLS which is followed by Triplex, 50 nt insert and AsCas12a crRNA against *CXCR4* flanked by direct repeats
pCMV-AsCas12a-trpl-ins_150nt#1-DR-cr1X4-DR	This study	Single plasmid encoding AsCas12a with 2xNLS which is followed by Triplex, 150 nt insert#1 and AsCas12a crRNA against *CXCR4* flanked by direct repeats
pCMV-AsCas12a-trpl-ins_150nt#2-DR-cr1X4-DR	This study	Single plasmid encoding AsCas12a with 2xNLS which is followed by Triplex, 150 nt insert#2 and AsCas12a crRNA against *CXCR4* flanked by direct repeats
pCMV-AsCas12a-trpl-DR-cr2X4-DR	This study	Single plasmid encoding AsCas12a with 2xNLS which is followed by Triplex and an array of two AsCas12a crRNAs against *CXCR4* flanked by direct repeats
pCMV-AsCas12a-trpl-DR-cr3X4-DR	This study	Single plasmid encoding AsCas12a with 2xNLS which is followed by Triplex and an array of three AsCas12a crRNAs against *CXCR4* flanked by direct repeats
pCMV-AsCas12a-trpl-4tDR-cr1X4-4tDR	This study	Single plasmid encoding AsCas12a with 2xNLS which is followed by Triplex and AsCas12a crRNA against *CXCR4* flanked by optimized direct repeats
pCMV-AsCas12a-trpl-4tDR-cr2X4-4tDR	This study	Single plasmid encoding AsCas12a with 2xNLS which is followed by Triplex and an array of two AsCas12a crRNAs against *CXCR4* flanked by optimized direct repeats
pCMV-AsCas12a-trpl-4tDR-cr3X4-4tDR	This study	Single plasmid encoding AsCas12a with 2xNLS which is followed by Triplex and an array of three AsCas12a crRNAs against *CXCR4* flanked by optimized direct repeats
pUC57Kan-trpl-6X4-6R5-3X43R5	This study, synthesized by Synbio (China)	Plasmid encoding three arrays of AsCas12a crRNA: an array of six crRNAs against *CXCR4*, an array of six crRNAs against *CCR5*, and an array of three crRNAs against *CXCR4* and three crRNAs against *CCR5*
pCMV-mClover-trpl-DR-cr6X4-DR	This study	Single plasmid encoding mClover which is followed by Triplex and an array of six AsCas12a crRNAs against *CXCR4* flanked by direct repeats
pCMV-mClover-trpl-DR-cr6R5-DR	This study	Single plasmid encoding mClover which is followed by Triplex and an array of six AsCas12a crRNAs against *CCR5* flanked by direct repeats
pCMV-mClover-trpl-DR-cr3X4_3R5-DR	This study	Single plasmid encoding mClover which is followed by Triplex and an array of three AsCas12a crRNAs against *CXCR4* and three AsCas12a crRNAs against *CCR5* flanked by direct repeats

pCMV-mClover-trpl-DR-cr3X4-DR	This study	Single plasmid encoding mClover which is followed by Triplex and an array of three AsCas12a crRNAs against *CXCR4* flanked by direct repeats
pCMV-mClover-trpl-DR-cr3R5-DR	This study	Single plasmid encoding mClover which is followed by Triplex and an array of three AsCas12a crRNAs against *CCR5* flanked by direct repeats
pCMV-FRB-AsCas12a-trpl-DR-cr1X4-DR	This study	Single plasmid encoding FRB-AsCas12a with 2xNLS and 3xHA which is followed by Triplex and AsCas12a crRNA against *CXCR4* flanked by direct repeats
pCMV-FRB-AsCas12a-trpl-DR-cr1R5-DR	This study	Single plasmid encoding FRB-AsCas12a with 2xNLS and 3xHA which is followed by Triplex and AsCas12a crRNA against *CCR5* flanked by direct repeats
pCMV-FRB-AsCas12a-trpl-DR-cr1Venus-DR	This study	Single plasmid encoding FRB-AsCas12a with 2xNLS and 3xHA which is followed by Triplex and AsCas12a crRNA against *Venus* flanked by direct repeats
pCMV-VSVG	#8454, Addgene [55]	Plasmid expressing VSVG used for VLP production
pHLS-FKBP12-Gag	#138476, Addgene [11]	Packaging plasmid for 'NanoMEDIC' VLP production
pHLS-FRB-SpCas9	#138477, Addgene [11]	Plasmid expressing FRB-SpCas9 for 'NanoMEDIC' VLP production
pCMV-BaEVRless	[56]	Plasmid encoding baboon endogenous retrovirus Rless glycoprotein (BaEVRless) used for VLP pseudotyping
pUCHR-inLuc-mR	[24, 57]	Plasmid encoding intron-regulated reporter vector inLuc

Supplementary Table S4. Summary of knockout levels induced by four types of AsCas12a-VLPs. Figures represent knockout levels (%, mean value ± standard deviation).

#	VLP design	*Venus*, 293-Venus clone #8	*CCR5*, 293T/CD4/CCR5 clone #19	*CXCR4*, Jurkat
50 µl dose				
1	VLPs #1<br>pCMV-AsCas12a<br>pKS-U6-crRNA	21,8 ± 9,94	18,1 ± 6,32	1,0 ± 0,2
2	VLPs #2<br>pCMV-FRB-AsCas12a<br>pKS-U6-crRNA	34,1 ± 11,5	27,7 ± 13,0	19,5 ± 0,7
3	VLPs #3<br>pCMV-FRB-AsCas12a<br>pCMV-mClover-trpl-DR-crRNA-DR	67,3 ± 14,3	90,5 ± 1,3	2,1 ± 1,0
4	VLPs #4<br>pCMV-FRB-AsCas12a-trpl-DR-crRNA-DR	45,9 ± 13,4	60,9 ± 12,6	0,9 ± 0,4
25 µl dose				
5	VLPs #1<br>pCMV-AsCas12a<br>pKS-U6-crRNA	12,8 ± 10,7	9,4 ± 5,3	0,4 ± 0,5
6	VLPs #2<br>pCMV-FRB-AsCas12a<br>pKS-U6-crRNA	20,8 ± 13,3	14,9 ± 11,8	0,4 ± 0,6
7	VLPs #3<br>pCMV-FRB-AsCas12a<br>pCMV-mClover-trpl-DR-crRNA-DR	45,1 ± 19,0	85,0 ± 5,1	10,7 ± 2,2
8	VLPs #4<br>pCMV-FRB-AsCas12a-trpl-DR-crRNA-DR	28,5 ± 17,4	41,7 ± 16,3	1,2 ± 0,4
12.5 µl dose				
9	VLPs #1<br>pCMV-AsCas12a<br>pKS-U6-crRNA	7,4 ± 6,7	3,4 ± 2,3	0,2 ± 0,3
10	VLPs #2<br>pCMV-FRB-AsCas12a<br>pKS-U6-crRNA	10,4 ± 9,4	7,7 ± 6,2	0,2 ± 0,3
11	VLPs #3<br>pCMV-FRB-AsCas12a<br>pCMV-mClover-trpl-DR-crRNA-DR	28,2 ± 20,0	72,2 ± 12,7	6,4 ± 1,2
12	VLPs #4<br>pCMV-FRB-AsCas12a-trpl-DR-crRNA-DR	16,7 ± 13,5	23,8 ± 11,2	0,6 ± 0,3

Supplementary References (correspond to reference numbers in the main text)

11. Gee, P.; Lung, M.S.Y.; Okuzaki, Y.; Sasakawa, N.; Iguchi, T.; Makita, Y.; Hozumi, H.; Miura, Y.; Yang, L.F.; Iwasaki, M.; et al. Extracellular nanovesicles for packaging of CRISPR-Cas9 protein and sgRNA to induce therapeutic exon skipping. *Nat. Commun. 2020 111* **2020**, *11*, 1–18, doi:10.1038/s41467-020-14957-y.

24. Maslennikova, A.; Kruglova, N.; Kalinichenko, S.; Komkov, D.; Shepelev, M.; Golubev, D.; Siniavin, A.; Vzorov, A.; Filatov, A.; Mazurov, D. Engineering T-Cell Resistance to HIV-1 Infection via Knock-In of Peptides from the Heptad Repeat 2 Domain of gp41. *MBio* **2022**, *13*, e0358921,

doi:10.1128/mbio.03589-21.

25. Chu, V.T.; Weber, T.; Wefers, B.; Wurst, W.; Sander, S.; Rajewsky, K.; Kühn, R. Increasing the efficiency of homology-directed repair for CRISPR-Cas9-induced precise gene editing in mammalian cells. *Nat. Biotechnol.* **2015**, *33*, 543–548, doi:10.1038/nbt.3198.

26. Chen, P.; Zhou, J.; Wan, Y.; Liu, H.; Li, Y.; Liu, Z.; Wang, H.; Lei, J.; Zhao, K.; Zhang, Y.; et al. A Cas12a ortholog with stringent PAM recognition followed by low off-target editing rates for genome editing. *Genome Biol. 2020 211* **2020**, *21*, 1–13, doi:10.1186/S13059-020-01989-2.

27. Liu, Z.; Liang, J.; Chen, S.; Wang, K.; Liu, X.; Liu, B.; Xia, Y.; Guo, M.; Zhang, X.; Sun, G.; et al. Genome editing of CCR5 by AsCpf1 renders CD4+T cells resistance to HIV-1 infection. *Cell Biosci. 2020 101* **2020**, *10*, 1–13, doi:10.1186/S13578-020-00444-W.

28. Kempton, H.R.; Goudy, L.E.; Love, K.S.; Qi, L.S. Multiple Input Sensing and Signal Integration Using a Split Cas12a System. *Mol. Cell* **2020**, *78*, 184-191.e3, doi:10.1016/J.MOLCEL.2020.01.016.

29. Gao, Z.; Herrera-Carrillo, E.; Berkhout, B. Improvement of the CRISPR-Cpf1 system with ribozyme-processed crRNA. *RNA Biol.* **2018**, *15*, 1458–1467, doi:10.1080/15476286.2018.1551703.

30. Kim, S.; Koo, T.; Jee, H.-G.; Cho, H.-Y.; Lee, G.; Lim, D.-G.; Shin, H.S.; Kim, J.-S. CRISPR RNAs trigger innate immune responses in human cells. *Genome Res.* **2018**, *28*, 367–373, doi:10.1101/gr.231936.117.

31. Cho, S.W.; Kim, S.; Kim, J.M.; Kim, J.-S. Targeted genome engineering in human cells with the Cas9 RNA-guided endonuclease. *Nat. Biotechnol.* **2013**, *31*, 230–232, doi:10.1038/nbt.2507.

32. Xin, C.; Yin, J.; Yuan, S.; Ou, L.; Liu, M.; Zhang, W.; Hu, J. Comprehensive assessment of miniature CRISPR-Cas12f nucleases for gene disruption. *Nat. Commun. 2022 131* **2022**, *13*, 1–10, doi:10.1038/s41467-022-33346-1.

33. Kleinstiver, B.P.; Sousa, A.A.; Walton, R.T.; Tak, Y.E.; Hsu, J.Y.; Clement, K.; Welch, M.M.; Horng, J.E.; Malagon-Lopez, J.; Scarfò, I.; et al. Engineered CRISPR-Cas12a variants with increased activities and improved targeting ranges for gene, epigenetic and base editing. *Nat. Biotechnol.* **2019**, *37*, 276–282, doi:10.1038/s41587-018-0011-0.

51. Ran, F.A.; Hsu, P.D.; Lin, C.-Y.; Gootenberg, J.S.; Konermann, S.; Trevino, A.E.; Scott, D.A.; Inoue, A.; Matoba, S.; Zhang, Y.; et al. Double nicking by RNA-guided CRISPR Cas9 for enhanced genome editing specificity. *Cell* **2013**, *154*, 1380–9, doi:10.1016/j.cell.2013.08.021.

52. Cong, L.; Ran, F.A.; Cox, D.; Lin, S.; Barretto, R.; Habib, N.; Hsu, P.D.; Wu, X.; Jiang, W.; Marraffini, L.A.; et al. Multiplex Genome Engineering Using CRISPR/Cas Systems. *Science (80-.).* **2013**, *339*, doi:10.1126/science.1231143.

54. Mali, P.; Yang, L.; Esvelt, K.M.; Aach, J.; Guell, M.; DiCarlo, J.E.; Norville, J.E.; Church, G.M. RNA-Guided Human Genome Engineering via Cas9. *Science (80-.).* **2013**, *339*, 823–826, doi:10.1126/science.1232033.

55. Stewart, S.A.; Dykxhoorn, D.M.; Palliser, D.; Mizuno, H.; Yu, E.Y.; An, D.S.; Sabatini, D.M.; Chen, I.S.Y.; Hahn, W.C.; Sharp, P.A.; et al. Lentivirus-delivered stable gene silencing by RNAi in primary cells. *RNA* **2003**, *9*, 493–501, doi:10.1261/rna.2192803.

56. Girard-Gagnepain, A.; Amirache, F.; Costa, C.; Lévy, C.; Frecha, C.; Fusil, F.; Nègre, D.; Lavillette, D.; Cosset, F.-L.; Verhoeyen, E. Baboon envelope pseudotyped LVs outperform VSV-G-LVs for gene transfer into early-cytokine-stimulated and resting HSCs. *Blood* **2014**, *124*, 1221–1231, doi:10.1182/blood-2014-02-558163.

57. Mazurov, D.; Ilinskaya, A.; Heidecker, G.; Lloyd, P.; Derse, D. Quantitative Comparison of HTLV-1 and HIV-1 Cell-to-Cell Infection with New Replication Dependent Vectors. *PLoS Pathog.* **2010**, *6*, e1000788, doi:10.1371/journal.ppat.1000788.
